# Volatile Organic Compounds Produced by *Trichoderma asperellum* with Antifungal Properties against *Colletotrichum acutatum*

**DOI:** 10.3390/microorganisms12102007

**Published:** 2024-10-03

**Authors:** Mauricio Nahuam Chávez-Avilés, Margarita García-Álvarez, José Luis Ávila-Oviedo, Irving Hernández-Hernández, Paula Itzel Bautista-Ortega, Lourdes Iveth Macías-Rodríguez

**Affiliations:** 1 Laboratorio de Bioquímica y Biología Molecular, División de Ingeniería Bioquímica, Tecnológico Nacional de México/ITS de Ciudad Hidalgo, Hidalgo 61100, Mexicoihernandez@cdhidalgo.tecnm.mx (I.H.-H.); pbautista@cdhidalgo.tecnm.mx (P.I.B.-O.); 2 Laboratorio de Bioquímica Ecológica, Instituto de Investigaciones Químico-Biológicas, Morelia 58030, Mexico; lmacias@umich.mx

**Keywords:** antifungal compounds, secondary metabolites, microbe interactions

## Abstract

Managing plant diseases caused by phytopathogenic fungi, such as anthracnose caused by *Colletotrichum* species, is challenging. Different methods have been used to identify compounds with antibiotic properties. *Trichoderma* strains are a source of novel molecules with antifungal properties, including volatile organic compounds (VOCs), whose production is influenced by the nutrient content of the medium. In this study, we assessed the VOCs produced in dual confrontation systems performed in two culture media by *Trichoderma* strains (*T. atroviride* IMI206040, *T. asperellum* T1 and T3, and *Trichoderma* sp. T2) on *Colletotrichum acutatum*. We analysed the VOC profiles using gas chromatography coupled with mass spectrometry. The Luria Bertani (LB) medium stimulated the production of VOCs with antifungal properties in most systems. We identified 2-pentyl furan, dimethyl disulfide, and α-phellandrene and determined their antifungal activity in vitro. The equimolar mixture of those VOCs (250 µM ea.) resulted in 14% *C. acutatum* diametral growth inhibition. The infective ability and disease severity caused by the mycelia exposed to the VOCs mixture were notably diminished in strawberry leaves. Application of these VOCs as biofumigants may contribute to the management of anthracnose. LB represents a feasible strategy for identifying novel VOCs produced by *Trichoderma* strains with antifungal properties.

## 1. Introduction

The *Colletotrichum* genus comprises approximately 600 species that cause anthracnose [1,2]. *Colletotrichum acutatum* and *C. gloeosporioides* are the most important species in the *Colletotrichum* complexes. These phytopathogens can affect different hosts, including tropical and subtropical fruits [3], and can cause significant economic losses of up to 50% [4]. The infection process of the *Colletotrichum* species begins with a biotrophic phase through the germination of resistance structures (appressoria and spores), which allows them to adhere to the host cell surface. The hyphae’s growth and development leads to intercellular and intracellular penetration, causing the infection to spread throughout the plant tissues. The necrotrophic phase begins once the hyphae of the pathogen are established in the host, and the development of secondary hyphae triggers the necrosis of adjacent plant cells. This cycle ends with the production of new reproductive structures [5,6,7]. This structure remains dormant when conditions are unfavourable for germination; therefore, controlling this structure is a complicated task [3]. Currently, various chemical methods are used to counteract anthracnose [8]. However, their excessive application induces resistance to phytopathogens [9], as well as the deterioration of consumer and producer health [10]. Therefore, alternative treatment options must be considered. The use of biological control agents (BCAs) is a strategy that aims to reduce the disease-producing activity caused by a pathogen through the application of one or more organisms [11]. BCAs are a source of different molecules with antimicrobial activity; for example, the *Trichoderma* species produce diffusible and volatile organic compounds (DOCs and VOCs, respectively) with antagonistic effects [12,13]. Furthermore, *Trichoderma* interacts with various microorganisms within the same microbial community through VOC emissions, which function as signalling molecules that allow them to modulate their metabolism according to the sensed population, creating a hostile environment for pathogen growth. Moreover, the diversity of antifungal VOCs produced by *Trichoderma* occurs in a strain-dependent manner [14], and their production can be affected by distinct factors, e.g., the chemical composition of the culture medium [15,16,17,18,19]. *Trichoderma harzianum*, *T. atroviride*, *T. hamatum*, *T. longibrachiatum*, *T. koningii*, *and T. viride* are among the *Trichoderma* species most commonly studied as BCAs. Recently, other species belonging to the Viride clade such as *T. asperellum* have gained attention [20]. The goal of the present study was to determine the *T. asperellum* strains’ ability to produce novel VOCs with fungicidal properties against *C. acutatum*, using dual confrontation systems in two culture media.

## 2. Materials and Methods

### 2.1. Molecular Identification of Trichoderma Strains

The *Trichoderma* strains were sent to the National Laboratory of Genomics for Biodiversity (LANGEBIO, Irapuato, México) for DNA extraction, PCR amplification, and sequencing. The nuclear DNA extraction was performed according to Lin et al. [21], for the PCR reactions, the primers ITS1 (5′-TCCGTAGGTGAACCTGCGG-3′) and ITS4 (5′- TCCTCCGCTTATTGATATGC-3′) were used [22]. The PCR products were bidirectionally sequenced using the corresponding primers by the Sanger method (Sequencing Standard, BigDye^TM^ Terminator v3.1, 3500/Flex Series, Applied Biosystems^TM^, Waltham, MA, USA) [23]. The sequences obtained were aligned against nucleotide sequences from the GenBank [24]. Additionally, the assembled sequences were deposited in Genbank under accession numbers PQ043841, PQ043842, and PQ043843.

### 2.2. Strains and Culture Conditions

The BCA strains used in this study were *T. atroviride* IMI206040, *T. asperellum* T1, *Trichoderma* sp. 2, *T. asperellum* T3, *C. acutatum*, and *Escherichia coli* (which was used as a negative control). All species were cultivated on potato dextrose agar (PDA, DIBICO^®^, Cuautitlán Izcalli, Edomex, Mexico) and Luria Bertani (LB) media at room temperature (21 ± 2 °C) in the dark for 10 d prior to bioassays.

### 2.3. Phylogenetic Analysis of Trichoderma Strains

To construct the phylogenetic trees of *T. asperellum* T1, *Trichoderma* sp. 2, and *T. asperellum* T3, the ITS sequences of *Trichoderma* strains and *Nectria eustromatica* were obtained from the GenBank database (Table 1). The downloaded sequences were aligned with SeaView v.5.05 using MUSCLE [25] and edited manually. The aligned sequences were used to perform Bayesian inference analysis in BEASTv.2.7.3 [26] using the TN93 substitution model and the estimated parameter priors with the Yule model. Three independent Markov chains of 10,000,000 replicates were run with sampling every 10,000. The sample parameters were combined with LogCombiner, and the basic parameters were checked using Tracer v.1.7.2 [27]. The sampled trees were combined with LogCombiner and then summarised using TreeAnnotator v.2.7.3. The posterior probability was calculated for each node in the maximum clade credibility tree with a burn-in of 10%. Phylogenetic trees were visualised using FigTree v.1.4.4. *N. eustromatica* was used to root the trees.

### 2.4. Antagonistic Activity of VOCs Produced by Trichoderma Strains against C. acutatum in Two Culture Media

The antagonistic activity of the *Trichoderma* strains against *C. acutatum* was evaluated using a dual confrontation system. For this bioassay, 20 mL of PDA medium was placed on Petri dishes (9 cm in diameter) and inoculated at the centre with 8 mm in diameter of colonised propagule from 10 d old culture of either antagonistic or phytopathogenic strains. The inoculated Petri bases were placed opposite each other and sealed with Parafilm^®^ [41]. This methodology allowed for the interaction between both microorganisms via VOC emissions. The dual confrontation systems were named as follows: *C. acutatum* vs. *Trichoderma* strains (*Cavs.*T1, *Cavs.*T2, *Cavs.*T3, and *Cavs.*IMI). In the bioassays performed on PDA medium, the phytopathogenic strain was inoculated first and *Trichoderma* strains (IMI206040, T1, T2, or T3) were inoculated 72 h later. In contrast, for the dual confrontation performed on LB medium, both *C. acutatum* and *Trichoderma* strains were inoculated simultaneously. Petri dishes inoculated with *C. acutatum* vs. *E. coli* were used as a negative control. The bioassays were incubated at room temperature (21 ± 1 °C) in darkness until the control saturated the base of the Petri dish or had no growth for 3 d in a row. The growth of the phytopathogenic strains was measured using a ruler each 24 h. Growth inhibition was determined using the following equation: growth inhibition ([(control growth − treated growth)/control growth] × 100) [42]. The experiments were repeated three times, with six replicates per treatment.

### 2.5. Identification of VOCs

The VOCs produced individually by all strains, as well as those produced in dual confrontations systems (*Cavs.*T1, *Cavs.*T2, and *Cavs.*T3) performed on LB medium, were analysed.

The confrontation systems were incubated at room temperature (21 ± 1 °C) in the dark for 5 d. The VOCs were collected using a blue solid-phase microextraction (SPME) fibre (PDMS/DVB) (Supelco, Inc., Bellafonte, PA, USA) and desorbed at 180 °C for 30 s in the injection port of a gas chromatograph (Agilent Technologies^®^ 7890 B GC system, Foster City, CA, USA) equipped with an MS detector 5973 from Agilent and a free fatty acid-phase capillary column (HP-FFAP) (30 m × 0.25 mm I.D., film thickness of 0.25 μm). Helium was used as the carrier gas (1 mL/min), and the detector temperature was 230 °C. The oven program was set at an initial temperature of 40 °C for 5 min, followed by a steady increase of 3 °C per min until a final temperature of 220 °C was reached, which was maintained for 5 min. The post-run temperature was set to 300° C for 3 min. The compounds were identified by comparison with the mass spectra from the NIST/EPA/NIH Mass Spectral Database 11 and the NIST Mass Spectral Search Program 2.0; Chemstation Agilent Technologies Rev. D.04.00 2002 [43]. Three independent measurements were made for each strain and confrontation system.

### 2.6. Analysis of the Antifungal Activity of the Synthetic VOCs Identified against C. acutatum

The antifungal activities of 2-pentyl furan, dimethyl disulfide, and α-phellandrene were evaluated individually against *C. acutatum*. A colonised propagule (8 mm in diameter) from 10 d old *C. acutatum* culture was placed on PDA medium at the centre of a Petri dish. Additionally, a filter paper disc (Whatman number four) was placed on the Petri dish lid with 0, 250, 500, and 1000 µM of each compound, individually. The inoculated Petri bases were placed on a Petri dish lid containing the compounds and were sealed with Parafilm^®^ (Amcor, Chicago, IL, USA), generating a head space volume of 60 mL. The head spaces of the Petri dishes were used to adjust the VOC concentrations. The assays were incubated at room temperature until the control strain saturated the base of the Petri dish or had no growth for 3 d in a row. Diametral growth was measured and the percentage of inhibition was plotted. The phytopathogen mycelia were observed using a Meiji Techno MX5300L Co. (Axbridge, UK) biological microscope equipped with a Meiji Infinit 1 metallographic camera with a 40× objective. Cell viability was evaluated by erioglaucine (Sigma-Aldrich, Waltham, MA, USA) staining with slight modifications [44].

### 2.7. Bioassays of Synthetic VOC Mixtures on the Growth and Development of C. acutatum

The concentrations that generated the most significant effects on the phytopathogen colonies were selected to formulate different mixtures. Bioassays were performed as described above and a mixture of synthetic VOCs was placed on a filter paper disc. The following VOC combinations were evaluated: α-phellandrene plus 2-pentyl furan (α-P+2-P), α-phellandrene plus dimethyl disulfide (α-P+DD), and α-phellandrene plus dimethyl disulfide plus 2-pentyl furan (α-P+DD+2-P). Each compound was assessed at 250 µM ea.

All bioassays described previously were incubated at room temperature (21 ± 1 °C) for 3 d in a row until the control saturated the base of the Petri dish or had no growth. Diametral growth was measured and the percentage of inhibition was plotted. At the end of the experiment, phytopathogenic mycelia were observed, as previously described.

### 2.8. Analysis of the Infectivity of the Mycelium of C. acutatum Exposed to Synthetic VOCs

Healthy strawberry leaves (*Fragaria* × *ananassa* Duch. Cv. Albion) were surface-disinfected with 1% Triton X-100 for 5 min, 70% ethanol for 1 min, and sodium hypochlorite for 10 min [45]. After washing three times in sterile deionised water, the leaves were transferred to a wet-chamber and inoculated with an 8 mm in diameter colonised propagule from the culture of *C. acutatum* exposed to the α-P+DD+2-P mixture as was previously described. A culture of *C. acutatum* without exposure to VOCs was used as a control. The leaves were incubated at room temperature (21 ± 1 °C) for 6 d in the dark. To analyse the infective activity of *C. acutatum* exposed to VOCs, propagules were withdrawn from the leaves and chemically treated for tissue clarification; leaves from each treatment were incubated for 2 h in a clearing solution (glacial acetic acid/ethanol (95%) 1:4 (*v/v*)) with continuous agitation. The clearing solution was replaced every hour until the tissue turned light yellow. The leaves were transferred to a 70% ethanol solution and incubated at 4 °C overnight, after which they were rinsed with sterile deionised water and incubated with 0.5 M EDTA until their evaluation. The leaves from each treatment were stained with erioglaucine (Sigma-Aldrich, MA, EE.UU.), and representative leaves from each treatment were chosen and imaged using Normaski optics on a Meiji Techno MX5300L Co. biological microscope equipped with a Meiji Infinit 1 metallographic camera with a 10× and 20× objectives.

### 2.9. Statistical Analysis

GraphPad Prism v.10.2.3 for Windows (Boston, MA, USA, www.graphpad.com, accessed on 2 August 2024) was used to perform statistical analyses. For the confrontation systems, synthetic VOCs antifungal activity, and severity evaluation, the data were expressed as the means ± SD of six repetitions. All data were analysed by one-way ANOVA followed by Tukey’s post hoc test. Differences were considered significant at α = 0.01. The principal component analysis (PCA) was performed using the factoextra package (version 1.0.7) and heatmap analysis was performed using the pheatmap package (version 1.0.12). Both analyses were performed with the R software (version 2024.04.2+764) (Posit team, 2024. RStudio: Integrated Development Environment for R. Posit Software, PBC, Boston, MA, USA, http://www.posit.co/, accessed on 2 August 2024) The VOCs’ abundance dataset was standardised previously to perform the multivariate analyses.

## 3. Results

### 3.1. Molecular Identification of Trichoderma Strains

**Figure 1:**
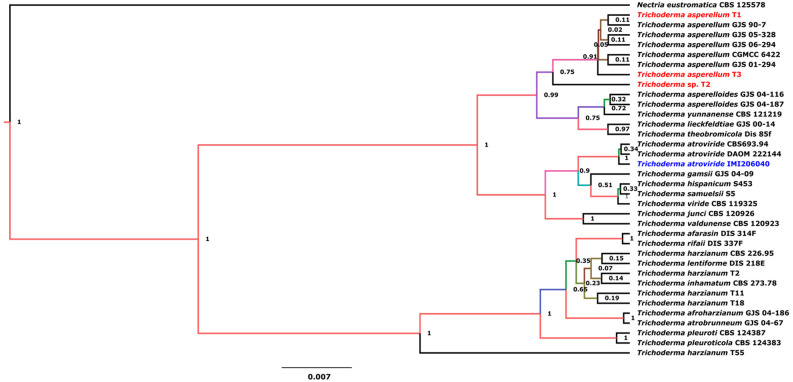
Bayesian tree inferred from ITS sequences of *Trichoderma* strains. Branch lengths are proportional to phylogenetic distance. The posterior probability values are shown in front of nodes. *Nectria eustromatica* was used to root the tree. The T1, T2, and T3 strains assessed in this work, as well as the reference strains (*T. atroviride* IMI206040), are shown in red and blue, respectively.

The most common species of the *Trichoderma* genus employed as BCAs include *T. harzianum*, *T. hamatum*, *T. longibrachiatum*, *T. koningii*, *T. viride*, and *T. polysporum.* Recently, research has focused on *T. asperellum* [23,24]. To identify the species of the *Trichoderma* strains used in this study, we performed molecular identification and phylogenetic analyses of the *Trichoderma* strains. Bayesian analysis of the ITS sequences situated the *Trichoderma* strains together in the clade *asperellum* with a posterior probability score of 0.99, although *Trichoderma* sp. T2 was separated from the group (Figure 1). This result indicated that the three *Trichoderma* strains used in this study corresponded to *T. asperellum*.

### 3.2. Antagonistic Activity of VOCs Produced by Trichoderma Strains against C. acutatum

Microbial VOCs with antagonistic effects have attracted attention; thus, BCA culture in different media is recommended to identify new molecules with antibiotic properties [5,25,26]. In this study, we determined the effect of culture media (PDA and LB) on the production of VOCs by BCAs (*T. atroviride* IMI206040, *T. asperellum* T1, *Trichoderma* sp. T2, and *T. asperellum* T3). In addition, we analysed the VOCs emitted during the confrontation between *C. acutatum* and *Trichoderma* strains, with the aim of identifying bioactive VOCs that inhibit the growth of the phytopathogen. For this purpose, the strain IMI206040 was used as a reference to estimate the effectiveness of *T. asperellum* strains.

Our results in the dual confrontation on PDA medium showed that the least efficient strains were T3 and T2, which inhibited the mycelial diameter growth of *C. acutatum* by 12.82% and 25.46%, respectively. In contrast, the most effective strains were IMI206040 and T1, with 47.41% and 42.14% inhibition, respectively (Table 2). Otherwise, we observed that the biocontrol activity of these strains changed in LB medium. The least competent was T1, with 33.96% diametral growth inhibition of *C. acutatum*. Moreover, the most effective strains were T2, T3, and IMI206040 with 56.86%, 51.84%, and 49.94% of diametral growth inhibition, respectively (Table 1). These results indicated that *T. asperellum* strains could be effective BCAs.

The results observed of the antagonistic bioassays suggested that the *Trichoderma’*s biocontrol activity is specific to each strain. This ability can be differentially influenced by factors such as nutrient sources, either by increasing or diminishing it.

### 3.3. Identification of VOCs

The LB culture medium produced the most significant changes in the inhibition of *C. acutatum* growth and morphology. These results suggested that the chemical composition of the medium favoured the production of volatiles with antifungal potential. Thus, the compounds produced individually and in the dual confrontation systems were determined using GC-MS.

**Figure 2:**
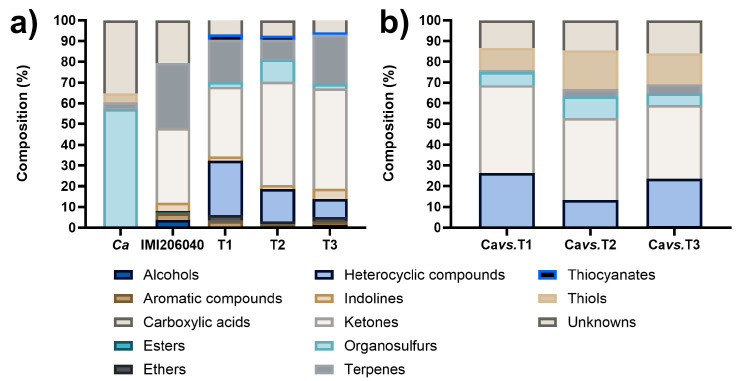
Composition of VOC profiles produced by the strains of study on LB medium. (**a**) Profiles of VOCs produced individually by the strains. (**b**) Profiles of VOCs produced in dual confrontation systems.

Under our experimental conditions, the total amount of VOCs emitted by the strains on LB medium were 10, 40, 34, 35, and 51 for *Ca*, IMI206040, T1, T2, and T3, respectively (Table S1). The VOCs were identified as alcohols, aromatics, carboxylic acids, esters, ethers, heterocyclic compounds, indolines, ketones, organosulfurs, terpenes, thiocyanates, thiols, and unknown compounds. The most abundant chemical classes for *Ca* were sulfur compounds and unknowns, 56.54% and 35.26%, respectively, while for *Trichoderma* strains, the VOCs’ composition was similar, but in different proportions: ketones (36.01%), terpenes (31.29%), and unknowns (20.57%) for IMI206040; ketones (33.44%), heterocyclic compounds (26.39%), and terpenes (20.38%) for T1; ketones (49.94%), heterocyclic compounds (15.58%), organosulfurs (10.79%), and terpenes (9.43%) for T2; and ketones (48.18%), terpenes (23.79%), and heterocyclic compounds (8.83%) for T3 (Figure 2a). The composition of the VOC profiles of *Trichoderma* strains reflects the diversity of chemical compounds produced by species of this genus. This represents a pool of compounds with antifungal potential that should be explored.

For dual confrontations, we assessed only the VOC profiles produced in the dual confrontation systems between *T. asperellum* strains and *C. acutatum* on LB medium. The identified VOCs mixture was principally composed of heterocyclic compounds, ketones, organosulfurs, thiols, and unknown compounds. The VOCs profile for the *Cavs.*T1 system was mainly composed of ketones (42.35%), heterocyclic compounds (26.36%), and unknown compounds (13.42%), while for the *Cavs.*T2 system, it was ketones (39.39%), thiols (18.78%), unknowns (14.45%), and heterocyclic compounds (12.79%); finally, the principal VOCs identified in the *Cavs.*T3 system were ketones (35.55%), heterocyclic compounds (22.76%), unknowns (15.92%), and thiols (14.98%) (Figure 2b). The number of VOCs detected in the dual confrontation systems was reduced compared to those produced individually, suggesting that the *Trichoderma* strains redirected their metabolism to produce antifungal compounds. Hence, compounds that integrate the principal chemical classes possess antifungal potential.

In this sense, the most abundant compounds identified for each chemical class were as follows: 6-Pentyl-2H-pyran-2-one from the ketones group with 41.55%, 38.16%, and 32.44% for *Cavs.*T1, *Cavs.*T2, and *Cavs.*T3, respectively; 2-pentylfuran from heterocyclic compounds with 20.44%, 3.47%, and 7.81%, respectively; methanethiol from thiols with 10.62%, 18.78%, and 14.98% for *Cavs.*T1, *Cavs.*T2, and *Cavs.*T3, respectively; finally, from the unknowns group, the compound with a retention time of 19.69 min was most abundant with 9.15%, 8.85%, and 10.90% for *Cavs.*T1, *Cavs.*T2, and *Cavs.*T3, respectively (Table 3). These compounds may be responsible for the antifungal activity against *C. acutatum*, although we did not exclude the possible contribution of other compounds to the inhibitory effect.

**Figure 3:**
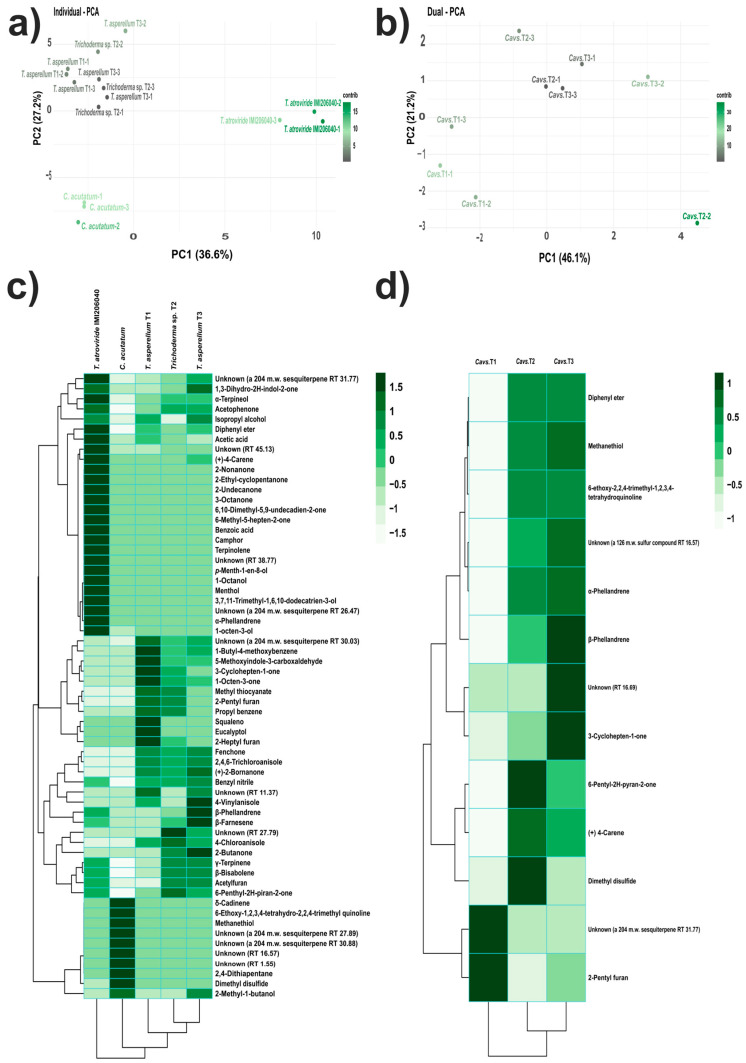
Multivariate analyses of VOCs produced on LB medium. (**a**,**b**) PCAs: (**a**) PC1 and PC2 of VOCs produced individually; (**b**) PC1 and PC2 of VOCs produced in dual confrontation systems; the variance explained by each principal component is reported in parenthesis. The scale bar indicates the contribution level of each system analysed to the establishment of principal components. (**c**,**d**) Heatmap and two-dimensional hierarchical dendrograms of VOCs produced by the strains: (**c**) individually, and (**d**) in dual confrontation systems. Each coloured cell on the map corresponds to the concentration value following a green chromatic scale from low to high production, where lighter colours correspond to smaller values and darker colours to larger values (scale bar).

The VOC profiles produced by the strains assessed in this study were similar but in different proportions. Hence, to identify the VOC patterns that differentiate the strains and dual confrontation systems according to their VOC profiles, we performed PCA. Thus, the individual VOC profiles of *T. asperellum* strains (T1, T2, and T3) were separated from *T. atroviride* IMI206040 and *C. acutatum* (Figure 3a). The two first principal components described 63.8% of the variation in the dataset.

Conversely, for the dual confrontation systems, the PCA highlighted the VOCs profile detected in *Cavs.*T1 from the other two systems analysed, while the profiles of *Cavs.*T2 and *Cavs.*T3 were grouped closely. The system with the major variation was *Cavs.*T2. The first two components accounted for 67.3% of the variation in the dataset (Figure 3b). These results suggested that strains corresponding to the *Trichoderma* genus possess versatile biosynthetic machinery, which represents a source of new molecules with possible antifungal potential.

The heatmap and two-dimensional hierarchical analysis of the individual VOC profiles on the LB medium showed defined clusters. The IMI206040 strain was separated from the rest of the strains analysed, as was the *Ca* strain, whereas the three *T. asperellum* strains were grouped closely. The IMI206040 strain overproduced twenty-five compounds, including 3-octanone, *p*-menth-1-en-8-ol, and unknown compounds (RT 38.77 and 45.13). For the *Ca* strain, all compounds detected were characteristic of this strain; hence, nine of them were overproduced, e.g., dimethyl disulfide and unknowns (RT 16.57 and 1.55). For the T1 strain, twelve compounds, such as 3-cyclohepten-1-one, 2-pentyl furan, squalene, and unknown (204 mW sesquiterpene TR 30.03), were overproduced. For the T2 strain, ten compounds were overproduced, e.g., unknown (RT 27.79), 2-pentyl furan, 4-chloroanisole, and 6-pentyl-2H-pyran-2-one. Finally, the T3 strain overproduced fifteen compounds, such as 4-vynilanisole, β-phellandrene, β-farnesene, 2-butanone, and 2-methyl-1-butanol, (Figure 3c).

In the dual confrontation systems, *Cavs.*T2 and *Cavs.*T3 integrated into a subgroup, whereas *Cavs.*T1 was separated from these. The overproduced compounds for dual confrontation systems were unknown (a 204 mW sesquiterpene RT 31.77) and 2-pentyl furan for the *Cavs.*T1 system. For the *Cavs.*T2 system, seven compounds were overproduced, among them 6-pentyl-2H-pyran-2-one, dimethyl disulfide, (+)-δ-carene, and α-phellandrene, while for the *Cavs.*T3 system, eight compounds were overproduced, including β-phellandrene, unknown (RT 16.69), 3-cyclohepen-1-one, α-phellandrene, and others (Figure 3d). The VOCs described above could be considered markers for each system analysed. In dual confrontation systems, those VOCs may have antifungal potential against *C. acutatum*.

### 3.4. Antifungal Activity of Synthetic VOCs against C. acutatum

#### 3.4.1. Antifungal Activity of Synthetic VOCs Individually Assessed against *C. acutatum* In Vitro

Three marker compounds from dual confrontation systems were selected for assessment of antifungal activity against *C. acutatum*. The selection criteria for these compounds were overproduction in dual confrontation systems, antifungal activity that had not been reported against *C. acutatum*, availability in the market, and accessibility. Under these criteria, the synthetic compounds selected were 2-pentyl furan, dimethyl disulfide, and α-phellandrene.

**Figure 4:**
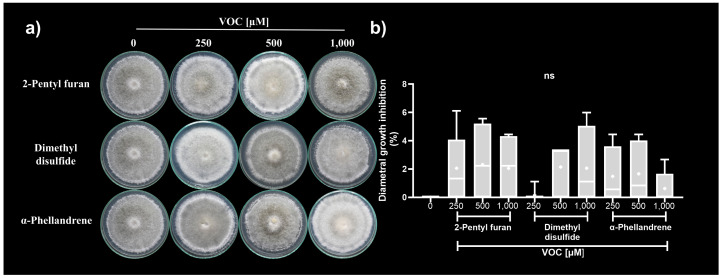
Antifungal activity of synthetic VOCs against *C. acutatum.* (**a**) Representative photographs from the *C. acutatum* colonies exposed to synthetic VOCs. (**b**) Diametral growth inhibition. The data in (**b**) represent the media of an *n* = 6 ± SD. ns indicates not statistically significant at one-way ANOVA followed by Tukey’s post hoc test (α = 0.01).

The synthetic VOCs assessed did not cause significant diametral growth inhibition of *C. acutatum* in any of the concentrations assessed (250, 500, and 1000 µM) (Figure 4a,b). Although the synthetic VOCs generated a discrete inhibitory effect on the phytopathogen, they caused similar alterations in the colony morphology (Figure 4a). The *C. acutatum* colonies exposed to synthetic VOCs individually developed lax colonies with altered pigmentation (gray to white). They showed a laxed aerial mycelium on the colony surface with sporulation rings on its edges. Additionally, the *C. acutatum* colonies’ exposure to 2-pentyl furan at 1000 µM developed vegetative mycelium on the colony border (Figure 4a).

In response to VOCs, the hyphae of *C. acutatum* showed different alterations in the three colony zones analysed (centre, middle, and edge). *C. acutatum* showed thinning hyphae at all concentrations of 2-pentyl furan assessed for all the samples analysed (Figure S1a). Similarly, dimethyl disulfide provoked curling, and vacuolisation, shortened hyphae, and distorted hyphae at 250 µM in the whole *C. acutatum* colony. The exposure to 500 µM dimethyl disulfide caused depolymerisation of the hyphae at the middle and edge zones from the colony. Additionally, this compound stimulated the *C. acutatum* sporulation in the middle zone from the colony at 1000 µM (Figure S1b). Finally, α-phellandrene induced sporulation at 250 µM on the entire colony, and thinned and curled hyphae, at higher concentrations in the distinct zones analysed (Figure S1c).

These results indicated that synthetic VOCs may have antifungal properties because these compounds alter hyphal development and pigmentation, which may diminish the infectious capacity of *C. acutatum*.

#### 3.4.2. Antifungal Activity of Synthetic VOC Mixtures against *C. acutatum* In Vitro

The synthetic VOCs generated common and specific microscopic alterations at different concentrations in *C. acutatum*. This suggested that VOCs have distinct targets that affect phytopathogen development. Furthermore, hyphal alterations such as vacuolisation, depolymerisation, and curling indicate damage to cellular processes such as hyphal polarised growth, cell-wall biosynthesis, and altered membrane potential. Hence, mixtures of these compounds may inhibit the growth of *C. acutatum*.

**Figure 5:**
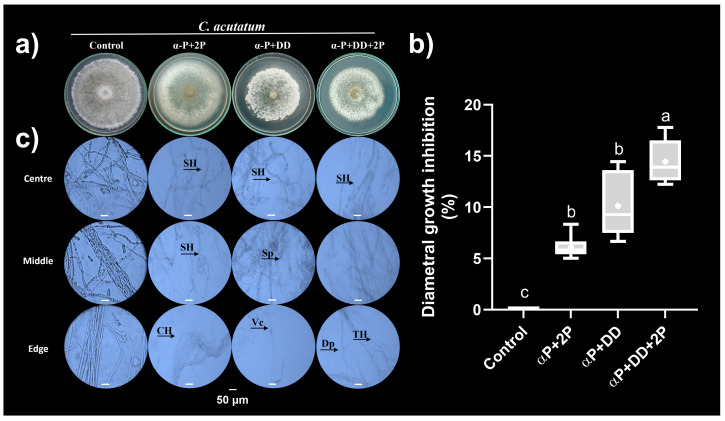
Antagonistic effect of the mixture of the synthetic VOCs on *C. acutatum.* (**a**) Representative photographs from the *C. acutatum* colonies exposed to synthetic VOC mixtures. α-P+2-P (α-phellandrene plus 2-pentyl furan); α-P+DD (α-phellandrene plus dimethyl disulfide); and α-P+DD+2-P (α-phellandrene plus dimethyl disulfide plus 2-pentyl furan); each compound was assessed at 250 µM. (**b**) Diametral growth inhibition. (**c**) Representative micrographs of mycelia of *C. acutatum* after 14 d of exposure to the VOCs’ mixtures described above. The mycelial samples were taken from three *C. acutatum* colony areas (centre, middle, and edge), mixed with a drop of brilliant blue, and visualised under a microscope with the 40× objective. TH (Thin Hyphae), CH (Curling Hyphae), Sp (Spores), SH (Swelling Hyphae), Vc (vacuolisation), and Dp (depolymerisation). Data in (**b**) are presented as the mean ± SD, *n* = 6. Different letters represent different statistically significant means (0.01 significance level in Tukey’s post hoc test).

Therefore, we assessed three mixtures of synthetic VOCs: α-phellandrene plus 2-pentyl furan (α-P+2-P), α-phellandrene plus dimethyl disulfide (α-P+DD), and α-phellandrene plus dimethyl disulfide plus 2-pentyl furan (α-P+DD+2-P); each compound was assessed at 250 µM ea. The combination of synthetic VOCs increased diametral growth inhibition of *C. acutatum.* The mixtures α-P+2-P, α-P+DD, and α-P+DD+2-P caused ~6%, ~10%, and ~14% diametral growth inhibition of *C. acutatum*, respectively (Figure 5a,b). This result indicated that VOCs had an additive effect on the diametral growth inhibition of *C. acutatum*.

Additionally, the morphology of the colonies showed more drastic alterations than those observed individually. The α-P+2-P mixture induced the development of white laxed mycelium at the centre of the colony’s fungal area; the effect described above was more evident when *C. acutatum* was exposed to the α-P+DD mixture as reflected by the formation of holes in the mycelium. Furthermore, this mixture induced the development of vegetative mycelia at the edges of colonies. The α-P+DD+2-P mixture had similar effects to those observed when *C. acutatum* was exposed to the α-P+DD mixture, but without the development of vegetative mycelia (Figure 5a).

At the microscopic level, the VOC mixtures induced alterations in hyphal development. The exposure of *C. acutatum* to the α-P+2-P mixture induced swelling and curling hyphae, while the α-P+DD mixture caused swelling hyphae, vacuolisation, and sporulation. Finally, the α-P+DD+2-P induced thinning and depolymerisation of the hyphae in addition to the effects described previously (Figure 5c).

These results reinforced the hypothesis that exposure of *C. acutatum* to VOC mixtures could affect the infective ability of phytopathogens.

### 3.5. Analysis of the Infectivity of the Mycelium of C. acutatum Exposed to the α-P+DD+2-P Mixture

Normal hyphal development and melanization are necessary for successful penetration of plant tissues by the phytopathogen. Since the principal alterations observed in the mycelia of *C. acutatum* exposed to the α-P+DD+2-P mixture included development of white colonies (decreased melanization) and alteration of the development of hyphae, we hypothesised that those fungal mycelia have diminished infective ability. To test this hypothesis, we determined the infectivity of *C. acutatum* using an ex vivo technique, employing leaves of strawberries *(Fragaria* × *ananassa* Duch. Cv. Albion, a host for phytopathogens).

**Figure 6:**
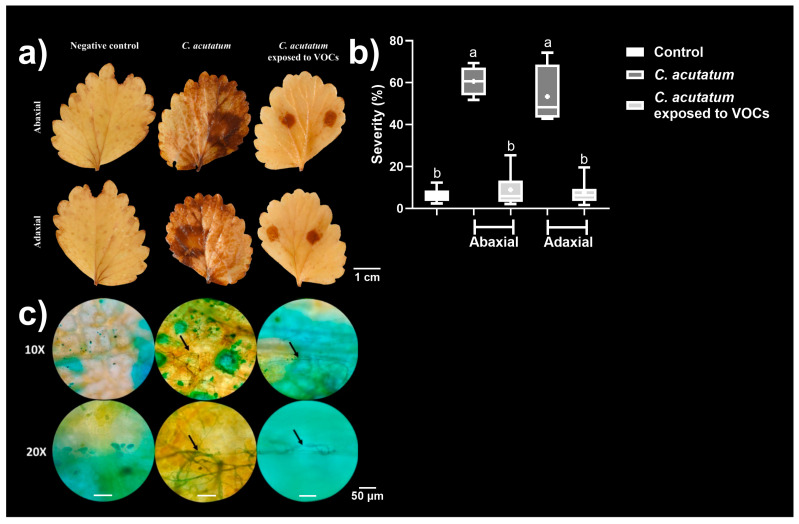
The infective ability of *C. acutatum* on strawberry leaves. (**a**) Representative photographs of strawberry leaves by the abaxial and adaxial sides. (**b**) Severity. (**c**) Representative micrographs of the hyphae of *C. acutatum* developed into the inner tissues of strawberry leaves. The samples were taken from the infected zones from strawberry leaves, mixed with a drop of erioglaucine, and visualised under a microscope with the 10× and 20× objective. The black arrows indicated the hyphal presence in the analysed zone. Data in (**b**) are presented as the mean ± SD, *n* = 6. Different letters represent different statistically significant means (0.01 significance level in Tukey’s post hoc test).

The untreated *C. acutatum* mycelium infected strawberry leaves and developed necrosis in both abaxial and adaxial tissues, with the infection severity reaching ~60% of the surface leaves. In addition, *C. acutatum* mycelium exposed to VOCs infected strawberry leaves, but in a less efficient manner than that developed by the untreated mycelia, and the infection was delimited to the contact zone with the propagule without developing necrosis, with the severity reaching <10% of the surface leaves (Figure 6a,b).

To verify the presence of the fungus in strawberry tissues, we performed a microscopic analysis, where the hyphae of *C. acutatum* were localised in the inner leaf tissues. Untreated hyphae of *C. acutatum* mycelium were more abundant than those exposed to VOCs (Figure 6c). These results indicated that the VOCs assessed in this study could contribute to diminishing infection caused by *C. acutatum*.

## 4. Discussion

The objective of this study was to determine the effects of two culture media (PDA and LB) on the *Trichoderma*´s biocontrol activity through the production of VOCs against *C. acutatum*, a cosmopolitan pathogen that causes anthracnose in economically important crops [46,47]. The diametral growth inhibition of *C. acutatum* in the dual confrontation system performed on LB was higher than that on PDA. The VOCs produced in the dual confrontation systems generated morphological alterations in the mycelia of pathogenic colonies. The *C. acutatum* colonies showed aerial mycelial growth, colony pigmentation changes, and irregular colony edge growth. These results are consistent with those reported by López-Hernández et al. [48], who suggested that the composition and abundance of VOCs produced in the different systems assessed are different and that they possess potential antifungal properties with distinct mechanisms of action [49].

The efficacy of the VOCs produced by *Trichoderma* strains on LB in inhibiting *C. acutatum* growth was probably due to the amino acids present [50]. Bruce et al. [51] demonstrated that the amino acid composition of the medium affects the production of fungicidal VOCs by *Trichoderma aureoviride*. Therefore, the use of different culture media with different nutritional compositions is a strategy to promote the production of secondary metabolites (SMs) with antifungal potential on *Trichoderma* strains.

Therefore, we analysed the VOCs produced individually by the strains and those produced in the dual confrontation systems on LB medium using GC-MS. Each individually assessed strain produced a different VOCs profile, both in terms of composition and abundance. Individually, *C. acutatum* mainly produced organosulfur and unknown compounds, whereas the most abundant VOCs produced by *T. atroviride* IMI206040 were ketones, terpenes, and unknown compounds. Conversely, *T. asperellum* T1, *Trichoderma* sp. T2, and *T. asperellum* T3 mainly produced ketones, terpenes, and heterocyclic compounds. This indicated that *Trichoderma* species possess a versatile metabolism based on the high number of genes involved in SM production [52]. Although the *Trichoderma* strains assessed in this study belonged to the same phylogenetic clade (*Trichoderma*), their VOC profiles showed notable differences, even between the *T. asperellum* strains, e.g., in the production of heterocyclic compounds, ketones, and terpenes. Hence, the VOC profile of *Trichoderma* was strain-dependent. These results are consistent with those reported in [14,15], which demonstrated that *Trichoderma harzianum*, *T. hamatum*, *T. reseei*, and *T. velutinum* strains produced specific VOC profiles.

*Trichoderma* species produce SMs with antimicrobial properties, including volatile and non-volatile molecules, which restrict the growth and development of other fungi. When *Trichoderma* strains were confronted with *C. acutatum*, the VOCs diversity was drastically reduced. This indicated that *Trichoderma* strains modulate their metabolism in response to fungal pathogens and other microorganisms. Guo et al. [15] reported that the VOC profiles of *Trichoderma harzianum*, *T. hamatum*, and *T. velutinum* were modulated (positively or negatively) when confronted with *Laccaria bicolor.*

Although the chemical diversity of the VOCs produced in the dual confrontation systems was similar in chemical classes, their abundance was different, e.g., for the *Cavs.*T1 system, the 2-pentyl furan abundance was 5.89 and 2.62 fold higher than those produced in the *Cavs.*T2 and *Cavs.*T3 systems, respectively; for the *Cavs.*T2 system, the dimethyl disulfide was 1.70 and 1.9 fold higher than those registered in *Cavs.*T1 and *Cavs.*T3 systems, respectively; finally, for the *Cavs.*T3 system, the 6-ethoxy-2,2,4-trimethyl-1,2,3,4-tetrahydroquinoline abundance was 2.52 and 1.6 fold higher than those produced in the *Cavs.*T1 and *Cavs.*T2 systems, respectively. These results suggested that *Trichoderma* strains produce VOC profiles in a strain-dependent manner in response to *C. acutatum*.

The multivariate analysis confirmed this assumption. The VOC profiles allowed us to discern the variation between *T. atroviride* IMI204060 and *T. asperellum* strains. Some compounds were identified as markers for each strain, as well as for the dual confrontation systems, which could have antifungal properties. *T. asperellum* T1, *Trichoderma* sp. T2, and *Trichoderma asperellum* T3 produced 6-pentyl-2H-pyran-2-one, and their antifungal activity has been reported against *Cylindrocarpon destructans* and *Peronophythora litchi*, which affects the amino acid metabolism and induces autophagy [53,54].

Therefore, we selected the marker VOCs identified in the dual confrontation systems and determined their antifungal properties against *C. acutatum*. We assessed the antifungal activity of 2-pentyl furan, dimethyl disulfide, and α-phellandrene against *C. acutatum*. None of the individually evaluated compounds significantly inhibited *C. acutatum* growth. However, they caused morphological alterations in the colonies, which induced the development of lax white mycelia. At the microscopic level, VOCs caused abnormal hyphal development, e.g., vacuolisation, distortion, thinning, and depolymerisation. Additionally, the α-phellandrene stimulated the *C. acutatum* sporulation. These effects indicated that *C. acutatum* faces stressful conditions in response to VOCs exposure. The diversity of alterations observed suggests that VOCs have different targets of action. Vacuolisation of hyphae indicates injury to the fungal cell-wall and plasma membrane, which triggers damage to the protoplasm and reduces cell viability [55]. The hyphae´s depolymerisation and distortion suggests affectations in the tubulin cytoskeleton, as this structure is an essential requirement for proper polarised growth; alterations in the formation of this cellular structure affect fungal morphogenesis and cause abnormal development of the hyphae [56]. Stimulated sporulation of *C. acutatum* may be associated with fungal survival [57].

Because the compounds assessed were identified as part of a VOCs blend in the dual confrontation systems, combining these compounds would generate an additive or synergistic effect on the growth inhibition of *C. acutatum*. In line with this, the *C. acutatum* diametral growth inhibition increased when it was exposed to the mixture α-P+DD+2P, reaching ~14% diametral growth inhibition. Additionally, microscopic alterations were more severe than those caused individually, causing hyphal swelling, depolymerisation, and thinning. These results reinforced the hypothesis that the VOCs mixture generates an additive effect and affects the same pathways but at different points.

Some monoterpenes can alter the plasma membrane, resulting in intracellular leaks derived from an increase in the cell membrane permeability of fungi [58,59]. Hence, it is hypothesised that α-phellandrene could cause damage to the cell membrane, allowing the internalisation of the other two VOCs and enhancing their toxic effects on *C. acutatum*. Zhang et al. [60] demonstrated that α-phellandrene provoke loss of cytoplasmic material and distortion of the mycelium in *Penicillium cyclopium*, causing an increase in their membrane permeability. The α-phellandrene potentiating effect was recently reported by Bhattacharya et al. [61], assessing it in combination with fluconazole and amphotericin B. Both combinations exerted synergistic effects against *Candida albicans*.

Moreover, Lin et al. [62] demonstrated the antifungal activity of dimethyl disulfide against *Magnaporthe oryzae*, *Gibberella fujikuroi*, *Sarocladium oryzae*, *Phellinus noxius*, *Colletotrichum fructicola*, and *Candida albicans.* Humphris et al. [63] reported that the ability of dimethyl disulfide to inhibit growth can be attributed to alterations in protein synthesis, which participates in fungal growth. Moreover, the 2-pentyl furan antifungal activity was demonstrated against *Monilinia fructicola* [64], *Sclerotinia sclerotiorum*, and *Fusarium oxysporum* [65]; however, its antifungal mechanisms have not been investigated. This molecule is classified as a heterocyclic compound; hence, it could share similar mechanisms of action with other heterocyclic compounds, e.g., inhibition of glucan synthesis [66].

In addition to microscopic alterations, the fungal colonies exposed to the VOCs mixture developed white mycelia. This indicated that melanin production in *C. acutatum* was diminished. Since hyphal melanization is required for appressoria to effectively penetrate plant tissues, we assessed their ability to infect strawberry leaves ex vivo. Exposure of *C. acutatum* to the VOCs mixture significantly diminished the disease severity caused by it on the strawberry leaves by approximately 85%. Laccases are responsible for melanin biosynthesis, and their production favours the pathogenicity of some fungi; hence, *C. acutatum’s* exposure to the VOCs mixture could have inhibited their activity [42].

## 5. Conclusions

Evaluation of the antagonistic activity of *T. asperellum* in different culture media (e.g., LB medium) represents a feasible strategy for identifying novel VOCs with antifungal properties, even between *Trichoderma* strains belonging to the same species. Moreover, the application of VOCs identified as biofumigants offers a strategy that could contribute to the management of plant diseases caused by fungi, such as anthracnose produced by *C. acutatum*. Additional research is necessary to determine whether the effectiveness of the VOCs identified in this study can be extrapolated to other *Colletotrichum* species.

## Figures and Tables

**Table 1:** Accession numbers for ITS sequences used for the phylogenetic tree.

Microorganism	Strain	GenBank Accession Number	Reference
*Trichoderma atroviride*	IMI206040	AF278795	[28]
*Trichoderma atroviride*	CBS693.94	KF576214	[29]
*Trichoderma atroviride*	DAOM 222144	AF456916	
*Trichoderma afarasin*	DIS 314F	FJ442259	
*Trichoderma atrobrunneum*	GJS 04-67	FJ442273	
*Trichoderma lentiforme*	DIS 218E	FJ442220	
*Trichoderma afroharzianum*	GJS 04-186	FJ442265	
*Trichoderma rifaii*	DIS 337F	FJ442621	
*Trichoderma asperelloides*	GJS 04-187	JN133553	[30]
*Trichoderma asperelloides*	GJS 04-116	GU198301	[31]
*Trichoderma asperellum*	GJS 05-328	GU198318	
*Trichoderma asperellum*	GJS 06-294	GU198307	
*Trichoderma asperellum*	GJS 90-7	GU198317	
*Trichoderma yunnanense*	CBS 121219	GU198302	
*Trichoderma asperellum*	GJS 01-294	EU856297	[32]
*Trichoderma asperellum*	CGMCC 6422	KF425754	[33]
*Trichoderma gamsii*	GJS 04-09	DQ315459	[34]
*Trichoderma harzianum*	T55	MW857216	
*Trichoderma harzianum*	T18	MW857216	[35]
*Trichoderma harzianum*	T2	OR794127	
*Trichoderma harzianum*	CBS 226.95	AY605713	
*Trichoderma harzianum*	T11	MT940829	
*Trichoderma hispanicum*	S453	JN715595	[36]
*Trichoderma samuelsii*	S5	JN715596	
*Trichoderma inhamatum*	CBS 273.78	MH861134	[37]
*Trichoderma pleuroti*	CBS:124387	MH863369	
*Trichoderma pleuroticola*	CBS:124383	MH863368	
*Nectria eustromatica*	CBS:125578	MH863715	
*Trichoderma junci*	CBS 120926	FJ860761	[38]
*Trichoderma valdunense*	CBS 120923	NR_134418	[39]
*Trichoderma viride*	CBS 119325	NR_138441	
*Trichoderma lieckfeldtiae*	GJS 00-14	DQ109528	[40]
*Trichoderma theobromicola*	Dis 85f	DQ109525	
*Trichoderma asperellum*	T1	PQ043841	This work
*Trichoderma* sp.	T2	PQ043842	
*Trichoderma asperellum*	T3	PQ043843	

**Table 2:** Antagonistic activity of *Trichoderma* strains against *C. acutatum* in different media.

Bioassay	*C. acutatum* Diametral Growth Inhibition (%)	
	Media	
	PDA	LB
Control (*E. coli*)	3.94 ± 3.35 c	1.23 ± 5.55 c
*T. atroviride* IMI206040	47.41 ± 5.77 a	49.94 ± 1.66 a
*T. asperellum* T1	42.14 ± 4.72 a	33.96 ± 9.23 b
*Trichoderma* sp. T2	25.46 ± 4.75 b	56.86 ± 2.60 a
*T. asperellum* T3	12.82 ± 3.71 c	51.85 ± 3.31 a

Note: Data are meant ± SD, *n* = 6. Different letters represent different statistically significant means by medium (PDA or LB) (0.01 significance level in Tukey’s post hoc test).

**Table 3:** VOCs produced in dual confrontation systems on LB medium.

Compound	Retention Time (min)	Abundance Relative (%)		
		*C. acutatum* vs. *T. asperellum* T1 (*Cavs.*T1)	*C. acutatum* vs. *Trichoderma* sp. T2 (*Cavs.*T2)	*C. acutatum* vs. *T. asperellum* T2 (*Cavs.*T3)
Methanethiol	0.93	10.62	18.78	14.98
3-Cyclohepten-1-one	2.73	0.80	1.23	3.11
Dimethyl disulfide	5.40	6.23	10.48	5.56
α-Phellandrene	8.53	-	1.26	1.49
(+)-4-Carene	9.16	-	1.89	2.00
β-Phellandrene	10.27	-	0.41	0.98
2-Pentylfuran	11.84	20.44	3.47	7.81
Unknown (a 126 m.w. sulfur compound)	16.57	4.27	5.60	5.02
Unknown	19.69	9.15	8.85	10.90
Unknown (a 204 m.w. sesquiterpene)	31.77	1.02	-	-
6-ethoxy-2,2,4-trimethyl-1,2,3,4-tetrahydroquinoline	37.30	5.92	9.32	14.95
Diphenyl ether	41.68	-	0.55	0.76
6-Pentyl-2H-pyran-2-one	46.90	41.55	38.16	32.44

Note: Compounds were tentatively identified based on NIST library searches.

## Data Availability

The data presented in this study was deposited into the GenBank database. The high-quality raw data genome sequence was deposited into NCBI under accession PQ043841-PQ043843.

## Supplementary Materials

The following supporting information can be downloaded at: https://www.mdpi.com/article/10.3390/microorganisms12102007/s1, Figure S1: Microscopic analysis of mycelium of *C. acutatum* exposed to synthetic VOCs; Table S1: VOCs produced by the fungal microorganisms on LB; Table S2: All data PCA loadings from VOCs produced by the fungal microorganisms on LB; Table S3: All data PCA loadings from VOCs produced on dual confrontation systems on LB.
