# Supplementary material for: Volatile Organic Compounds Produced by Trichoderma asperellum with Antifungal Properties against Colletotrichum acutatum
Source: Microorganisms. 2024 Oct 3;12(10):2007. doi: 10.3390/microorganisms12102007 (PMC11509848; doi:10.3390/microorganisms12102007)
Supplement: Supplementary file 1 [file microorganisms-12-02007-s001.zip › Figure S1.pdf]

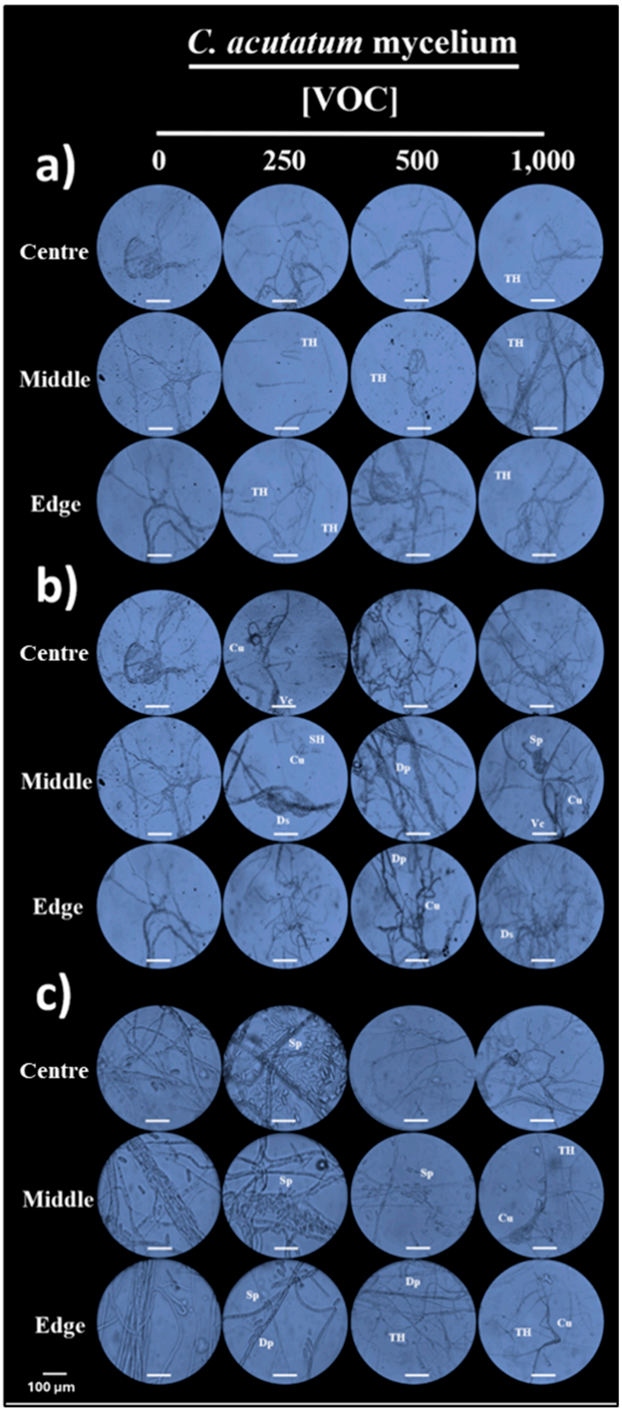

Figure S1. Microscopic analysis of mycelium of *C. acutatum* exposed to synthetic VOCs. The figure shown the representative micrographs of mycelium of *C. acutatum* after 13 to 15 d of exposition to VOCs at 0 µM, 250 µM, 500 µM, and 1,000 µM, of each compound assessed individually. **(a)** 2-pentyl furan, **(b)** dimethyl disulfide, and (**c**) α-phellandrene. The mycelial samples were taken from three *C. acutatum´* colony areas (center, middle, and edge) and mixed with a drop of erioglaucine and

visualized under a microscope. Scale bar represents 100 μM. TH (Thin Hyphae), Vc (Vacuolization), Ds (Distortion), Dp (Depolymerization), CH (Curling Hyphae), SH (Swelling Hifae), and Sp (Spores).
