# Supplementary material for: Volatile Organic Compounds Produced by Trichoderma asperellum with Antifungal Properties against Colletotrichum acutatum
Source: Microorganisms. 2024 Oct 3;12(10):2007. doi: 10.3390/microorganisms12102007 (PMC11509848; doi:10.3390/microorganisms12102007)
Supplement: Supplementary file 1 [file microorganisms-12-02007-s001.zip › Table S1. VOCs produced by the fungal microorganisms on LB.pdf]

Compound	Retention time (min)	Abundance relative (%)				
		C. acutatum	*T. atroviride* IMI206040	*T. asperellum* T1	*Trichoderma* sp. T2	*T. asperellum* T3
Methanethiol	0.93	4.30	-	-	-	-
Unknown	1.55	6.50	-	-	-	-
Isopropyl alcohol	2.11	-	0.39	0.48	-	0.49
2-Butanone	2.12	-	-	-	0.44	0.72
3-Cyclohepten-1-one	2.56	-	-	5.54	2.08	1.17
2-Pentanone	2.96	-	0.19	-	-	0.07
Dimethyl disulfide	4.38	56.08	-	2.35	10.25	1.79
2-Methyl-1-propanol	6.14	-	-	-	-	0.05
α-Phellandrene	7.70	-	2.30	-	-	0.14
1-Butanol	8.20	-	-	-	-	0.06
(+)-4-Carene	8.30	-	1.25	-	-	0.26
1,4-Cineol	9.08	-	-	-	-	0.20
β-Phellandrene	9.40	-	4.70	-	1.10	9.63
2-Heptanone	9.52	-	0.42	-	-	0.20
2-Methyl-1-butanol	10.11	0.49	-	-	-	0.27
Eucalyptol	10.19	-	-	1.08	-	0.16
Propyl benzene	10.52	-	-	1.10	0.55	0.31
Unknown	11.37	-	-	4.56	3.09	2.72
2-Pentyl furan	11.81	-	-	25.63	14.91	8.16
γ-Terpinene	11.94	-	0.22	-	-	-
2,4-Dithiapentane	12.73	0.46	-	-	-	-
3-Octanone	12.74	-	6.59	-	-	-
Methyl thiocyanate	13.84	-	-	2.57	1.91	0.94
2-Ethyl cyclopentanone	13.88	-	0.48	-	-	-
2-Octanone	14.26	-	-	-	-	0.39
1-Octen-3-one	15.03	-	-	0.65	0.24	0.23
Unknown	16.57	28.76	-	-	-	-
6-Methyl-5-hepten-2-one	16.62	-	0.86	-	-	-
Dimethyl trisulfide	16.77	-	-	-	0.54	0.46
Fenchone	18.59	-	-	0.39	0.22	0.34
2-Nonanone	18.80	-	0.49	-	-	-
2-Heptyl furan	20.76	-	-	0.76	0.09	-
p-Menthan-3-one	21.48	-	-	-	0.23	0.32

Compound	Retention time (min)	Abundance relative (%)				
		C. acutatum	***T. atroviride* IMI206040**	***T. asperellum* T1**	***Trichoderma* sp. T2**	***T. asperellum* T3**
1-octen-3-ol	21.68	-	0.93	0.27	0.15	0.14
Acetic acid	21.98	-	0.45	0.27	0.18	0.13
1-Octanol	22.32	-	1.45	-	-	-
Camphor	23.45	-	0.57	-	-	-
(+)-2-Bornanone	23.47	-	-	0.74	0.38	0.67
2-Acetylfuran	23.96	-	0.34	-	0.58	0.67
Terpinolene	25.79	-	1.22	-	-	-
Unknown (a 204 m.w. sesquiterpene)	26.47	-	0.81	-	-	-
Menthol	26.70	-	0.99	-	-	-
Neomenthol	27.29	-	-	0.28	0.21	0.13
2-Undecanone	27.41	-	1.14	-	-	-
Unknown	27.79	-	-	2.28	2.62	1.57
Unknown (a 204 m.w. sesquiterpene)	27.89	1.16	-	-	-	-
4-Chloroanisole	28.66	-	-	0.67	0.85	0.56
1-Butyl-4-methoxybenzene	28.85	-	-	0.90	0.21	0.38
Acetophenone	29.38	-	1.43	1.25	1.30	1.65
Unknown (a 204 m.w. sesquiterpene)	30.03	-	0.70	10.17	3.54	4.61
δ-Cadinene	30.29	0.93	-	-	-	-
p-Menth-1-en-8-ol	30.36	-	15.41	-	-	-
γ-Terpinene	30.37	-	-	0.17	0.34	0.39
4-Vinylanisole	30.54	-	-	0.36	-	0.68
Unknown (a 204 m.w. sesquiterpene)	30.88	1.08	-	-	-	-
α-Terpineol	31.13	-	0.86	0.50	0.65	0.69
Unknown (a 204 m.w. sesquiterpene)	31.77	-	1.54	0.31	0.53	1.18
β-Bisabolene	31.98	-	0.62	0.50	0.91	1.02
α-Farnesene	33.06	-	-	-	-	0.08
1,3-Dihydro-2H-indol-2-one	33.24	-	3.72	0.15	1.19	4.36
β-Farnesene	33.48	-	1.00	-	1.32	3.97
Methyl salicylate	33.88	-	0.39	-	-	0.27
2,4,6-Trichloroanisole	34.98	-	-	0.53	0.30	0.29
2-Naphthanelol	35.89	-	-	-	-	0.06
6,10-Dimethyl-5,9-undecadien-2-one	36.76	-	1.22	-	-	-

Compound	Retention time (min)	Abundance relative (%)				
		C. acutatum	*T. atroviride* IMI206040	*T. asperellum* T1	*Trichoderma* sp. T2	*T. asperellum* T3
6-Ethoxy-1,2,3,4-tetrahydro-2,2,4-Trimethylquinoline	37.30	0.24	-	-	-	-
Unknown	38.77	-	12.25	-	-	-
Benzyl nitrile	39.26	-	0.32	0.77	0.61	0.80
Diphenyl eter	41.67	-	0.45	0.42	0.24	0.31
3,7,11-Trimethyl-1,6,10-dodecatrien-3-ol	43.01	-	0.52	-	-	-
p-Cresol	44.45	-	0.22	0.22	-	0.03
1H-Indol-4-ol	44.57	-	0.35	-	-	0.04
Unkown	45.13	-	8.32	-	1.77	1.91
6-Pentyl-2H-pyran-2-one	46.90	-	22.67	26.00	45.88	43.75
β-Elemene	48.47	-	-	0.57	-	-
Benzoic acid	54.73	-	2.06	-	-	-
5-Methoxyindole-3-carboxaldehyde	56.70	-	-	1.92	0.59	0.58
Squalene	67.54	-	0.14	5.67	-	-

Note: Compounds were tentatively identified based on NIST library searches.
